# Therapeutic arts in Alzheimer disease: a brief review of evidence and mechanisms

**DOI:** 10.1590/1980-5764-DN-2025-0467

**Published:** 2026-10-12

**Authors:** Sristi Chakraborty

**Affiliations:** 1 Manipal Academy of Higher Education, Kasturba Medical College – Manipal Karnataka, India.

**Keywords:** Alzheimer Disease, Art Therapy, Music Therapy, Dance Therapy, Cognitive Dysfunction, Doença de Alzheimer, Arteterapia, Musicoterapia, Dançaterapia, Disfunção Cognitiva

## Abstract

As pharmacological treatments for Alzheimer disease (AD) offer only modest symptomatic improvement, non-pharmacological interventions — including therapeutic arts — are increasingly being explored for their potential to enhance cognitive, emotional, and psychosocial well-being in individuals with AD. This mini-narrative review aimed to summarize the various neurobiological mechanisms through which therapeutic arts impact progression of AD. It also compares the findings, clinical benefits, and changing attitudes of patients and caregivers toward therapeutic arts, such as music therapy, visual arts, dance/movement therapy, and creative expression, before and after 2000. With robust studies and advanced technologies, there is growing evidence that integrating structured arts-based programs into dementia care may provide cost-effective solutions to improve patient outcomes and slow functional decline in patients with AD.

## RESUMO

Como os tratamentos farmacológicos para a doença de Alzheimer (DA) proporcionam apenas uma melhora sintomática modesta, intervenções não farmacológicas – incluindo as artes terapêuticas – têm sido cada vez mais investigadas devido ao seu potencial para promover o bem-estar cognitivo, emocional e psicossocial de indivíduos com DA. Esta mini revisão narrativa tem como objetivo sintetizar os diversos mecanismos neurobiológicos pelos quais as artes terapêuticas influenciam a progressão da DA. Também compara as evidências, os benefícios clínicos e a mudanças nas atitudes de pacientes e cuidadores em relação às artes terapêuticas, como musicoterapia, artes visuais, dança/movimento terapêutico e expressão criativa, antes e após o ano 2000. Com a disponibilidade de estudos robustos e tecnologias avançadas, há evidências crescentes de que a integração de programas estruturados baseados em artes ao cuidado de pessoas com demência pode representar uma estratégia custo-efetiva para melhorar os desfechos clínicos e retardar o declínio funcional em pacientes com DA.

## INTRODUCTION

Incorporating music and the arts in daily life has been shown to be effective in managing the symptoms of multiple mental health disorders in the long term^1^. Although the exact protective mechanisms remain incompletely understood, it is hypothesised that therapeutic arts engage sensory, emotional, motor, attentional, and memory-related networks simultaneously and are more likely to enhance neural plasticity in both sensory and association areas of the brain during the earlier stages of the disease^2^. Extensive research investigating whether active participation in the arts decreases the incidence of Alzheimer disease (AD) and dementia is being conducted through longitudinal cohort studies, randomized controlled trials (RCTs), and neuroimaging studies^3^. Since cognitive decline begins long before clinical diagnosis^4^, interest in early non-pharmacological interventions, such as therapeutic arts, continues to rise.

In the context of AD, subjective cognitive decline (SCD) — self-reported cognitive lapses without objective clinical impairment^5^ — is increasingly recognized as a vital preclinical stage for early intervention. Since the role of pharmacological interventions is limited, the current focus has shifted toward non-pharmacological interventions. Various forms of art therapy are being explored, including music, singing, dancing, reading and poetry groups, museum/gallery art and collections, creative writing, life story narrative reminiscence, painting, printmaking, collage, pottery, sewing, knitting, woodwork, gardening^6^, and others. Art therapy is strongly recommended for older adults with complex neurological symptoms when pharmacotherapy can no longer alleviate the symptoms^7,8^.

Studies conducted across Europe and the USA have been shown to be widely beneficial in clinical practice^9^. It has been suggested that music therapy can alleviate both subjective and objective memory decline in symptomatic adults^10^. In contrast, dance therapy improves episodic memory and processing speed while preserving muscle memory and coordination^11^. The positive effects of therapeutic gardening on social, psychological, and cognitive health, has also been mentioned in a narrow review^12^. Spiritual reminiscence programs using expressive arts therapy have been shown to improve cognitive function^13^. Expressive arts therapy (EAT) involves information processing across four dimensions: cognitive/symbolic, perceptual/affective, kinesthetic/sensory, and creative. Simultaneous integration across these domains helps arouse individuals’ curiosity and improve sensation, orientation, memory, visuospatial function, and hand-eye coordination, while strengthening verbal interactions with others^14^.

Despite growing interest in therapeutic arts for dementia care, the evidence base has developed unevenly over time. Early investigations were exploratory, whereas contemporary work has adopted increasingly structured and mechanistic approaches. To contextualize the strengths and limitations of the current literature, this review includes a brief historical comparison of studies published before 2000 and those published between 2000 and 2024.

## METHODS

In the context of this review, the term “therapeutic arts” is used as an umbrella concept encompassing a range of structured and unstructured creative interventions, including music therapy, visual arts, dance or movement-based activities, and storytelling, highlighting their shared goal of enhancing cognitive, emotional, and behavioral outcomes in individuals with AD.

A non-systematic search of PubMed, Scopus, and PsychINFO was conducted for articles published between 2000 and 2024. Relevant literature was identified using combinations of keywords such as “Alzheimer’s disease,” “therapeutic arts,” “music therapy,” “art therapy,” “dance therapy,” and “creative arts therapy”. Articles were selected based on their relevance to clinical outcomes and neurobiological mechanisms of arts-based interventions in AD. Priority was given to randomized controlled trials and observational studies.

The same search strategy was applied to select foundational studies published prior to 2000 to provide historical context and illustrate early exploratory approaches to therapeutic arts. These studies were chosen based on their citation frequency, indexing, peer-review status, relevance to early clinical applications, and representation of key modalities.

Given the narrative nature of this review, no formal inclusion or exclusion criteria, systematic screening process, or quantitative synthesis was applied.

### Historical evidence for therapeutic arts in Alzheimer disease

Early investigations into therapeutic arts in dementia were largely exploratory and focused predominantly on music-based interventions, partly due to their relative ease of implementation in institutional settings. During the late 1980s and 1990s, most studies consisted of small pilot trials, observational designs, or uncontrolled clinical interventions aimed at assessing patient engagement, mood, and behavioral responses rather than formal cognitive outcomes, as summarized in Table 1. These foundational studies, though limited by small sample sizes, short intervention periods, and non-standardized outcome measures, played a key role in shaping later interest in non-pharmacological interventions for AD.

**Table 1:** Selected pre-2000 studies on therapeutic arts in dementia.

Study (Year)	Design/Sample	Intervention	Primary Outcome(s)	Key Message
Clair and Bernstein (1990)^15^	Pilot case series of 3 men with severe AD	Weekly 30-minute music sessions for 15 months	Engagement and functioning in groups activities regardless of cognitive deterioration.	Feasible in severe AD; improved engagement.
Lord and Garner (1993)^16^	Controlled clinical study of 60 patients with AD	Divided into and assigned to 3 groups: “band music,” puzzles, and drawing during the daily recreation period	Alertness, mood, and autobiographical recall were higher in patients assigned to the “band music” group.	Music group showed improved mood and recall vs. controls.
Groene (1993)^17^	1:1 intervention trial of 30 patients with AD	Assigned to either five music and two reading sessions or five reading and two music sessions	Participants remained seated in close proximity to the music session area for longer than to the arts session area.	Improved attentional response to music sessions vs. arts sessions.
Pollack and Namazi (1992)^18^	Controlled clinical study of 8 patients with AD	Six 20-minute music therapy sessions over 2 weeks.	Increased participation, smiling, eye contact, and verbal feedback expressing pleasure	Increased social behavior
Standley (1996)^19^	Meta-analysis (broader field) of 98 studies	Music as a contingency for reinforcement/therapy	Overall benefits under contingent music were 3SD higher vs. controls	Supported the therapeutic use of music across settings.
Brotons and Pickett-Cooper (1997)^20^	Literature review of 30 empirical clinical studies (1985–1996)	Heterogenous interventions	Improved social, cognitive, and behavioral outcomes associated with music	Music may be a medium for older adults with AD to communicate and access memories.
Kumar et al. (1999)^21^	Physiological pilot study	Blood biomarker measurement following 4 weeks of music therapy sessions	Increased serum melatonin after music therapy sessions	Suggested a biological mechanism for calming and sleep effects.

Abbreviation: AD, Alzheimer disease.

### Present neurobiological rationale for therapeutic arts

Therapeutic arts engage distributed neural networks involved in cognition, emotion, and sensorimotor integration. However, much of the neurobiological rationale for their use in AD is derived from a combination of limited patient-based studies and broader findings from cognitive neuroscience and animal model research.

#### Multimodal network activation

Neuroimaging studies in individuals with AD suggest that certain artistic modalities, particularly long-familiar music^22^, activate multiple brain regions simultaneously, including the auditory cortex, visual cortex, premotor areas, prefrontal cortex, and limbic system^23^. Functional imaging studies have also demonstrated activation of areas such as the anterior cingulate cortex and medial prefrontal cortex, which may remain partially intact even in the early stages of cognitive decline^24^. This multimodal stimulation supports residual cognitive reserves in AD and encourages stronger neural engagement.

#### Hippocampal neuroplasticity

Mechanisms such as enhanced synaptic plasticity and increased connectivity are largely extrapolated from studies in aging individuals and animal models^25,26^. While studies in neurorehabilitation have shown that creative tasks promote synaptic plasticity^27^ in emotional and sensory circuits in the hippocampus, direct evidence demonstrating similar effects in AD remains limited. Therefore, claims regarding synaptic remodeling and network-level reorganization in AD should be interpreted cautiously.

#### Emotional memory preservation

Emotional memory processing, particularly in relation to music, overlaps with areas that are relatively spared early in the disease process^28^, such as the anterior cingulate cortex and ventral pre-supplementary motor area. Engaging this preserved emotional circuitry has been associated with increased emotional positivity and self-reliance^29^. These approaches may facilitate the recall of emotionally salient memories, even when declarative memory is significantly impaired. However, much of this evidence is derived from small clinical studies and observational findings, and the underlying mechanisms remain incompletely understood.

#### Reward pathway activation

Engaging in meaningful creative activities triggers dopaminergic pathways, particularly in response to creative expression. While this may contribute to improvements in mood and motivation, decrease agitation, and enhance calmness^23^, most supporting evidence is derived from general neuroscience and affective studies rather than AD-specific populations^30^, and research involving AD-specific neuroimaging is needed to establish causal relationships and clarify the extent of these effects.

### Modern-day modalities for therapeutic arts and evidence

Therapeutic arts interventions can be broadly categorized based on their mode of engagement — receptive modalities (*e.g*., music listening), active or expressive modalities (*e.g*., painting, storytelling), and motor-based interventions (*e.g*., dance and movement therapies). These categories often overlap in practice but provide a useful framework for understanding their underlying mechanisms and clinical applications.

#### Music therapy

In comparison with other therapeutic arts modalities, music therapy is the most extensively studied in AD, as supported by multiple RCTs and systematic reviews^31,32,33^. Some studies have reported modest improvements in autobiographical recall^34^, agitation, mood, and cognition^35^ following exposure to familiar tunes and childhood melodies; however, these findings are less consistent across trials. Heterogeneity across intervention protocols, duration, and outcome measures further limits direct comparison across studies.

#### Visual arts (painting, drawing, sculpting)

Visual arts-based interventions have been associated with improvements in emotional well-being and self-expression in individuals with AD^36^. Observational studies suggest that certain visuospatial and creative abilities may be relatively preserved in the early to moderate stages^30^, and report improved mood, engagement, and reduced apathy. However, evidence for direct cognitive benefits and measurable cognitive outcomes remains limited and inconsistent.

#### Dance and movement-based therapy

Dance therapy integrates rhythmic movement, music, and physical exercise and has been associated with improvements in balance, coordination, and overall well-being. Some studies suggest movement-based interventions promote neuronal activation and enhanced connectivity across frontal, limbic, and cerebellar regions^37,38^; reinforcing neuroplasticity^39^. This evidence is again derived from small or heterogeneous studies, and direct evidence supporting these mechanisms in AD remains limited. Additionally, substantial variability exists in intervention design, including differences in intensity, structure, and duration, which complicates the interpretation and generalizability of the findings.

#### Storytelling, poetry, and creative expression

Narrative therapies allow individuals to share experiences, maintain identity, and engage language networks. Van der Weel and Van der Meer^40^ discovered that writing by hand increases brain connectivity, which has been shown to facilitate learning and memory. Although most of the literature on the “production effect” comes from studies involving healthy individuals, a few reports have suggested improved memory recall and executive function in AD, as well as the possibility of using it as a screening tool to detect preclinical AD^41,42^.

### Comparative synthesis of evidence across modalities

Across therapeutic arts modalities, the strength and nature of evidence vary considerably in terms of study design, outcomes assessed, and consistency of findings. Music-based interventions remain the most widely studied in AD, with consistent evidence demonstrating improvements in behavioral and psychological symptoms. However, cognitive benefits require further evidence from large-scale trials.

In contrast, visual arts, dance- and movement-based, and storytelling interventions are supported predominantly by smaller-scale studies. While these modalities show promising effects on emotional well-being, engagement, and quality of life, evidence for sustained cognitive improvement is more limited and heterogeneous.

While behavioral outcomes show relatively consistent improvement across studies, cognitive outcomes demonstrate variable and less robust effects. Additionally, substantial heterogeneity exists in intervention protocols, duration of exposure, and outcome measures, limiting direct comparison across modalities.

Overall, while therapeutic arts interventions appear beneficial as adjuncts for improving quality of life and behavioral symptoms in AD, further large-scale, standardized randomized trials are required to clarify their impact on cognitive trajectories and establish modality-specific effectiveness.

### Evaluation of evidence and perspectives across decades

Taken together, the historical literature shows a clear progression from small behavioral observations to structured interventions supported by validated scales and, more recently, neurobiological evidence. Rather than repeating individual findings, the comparison illustrated in Table 2 highlights a shift in study design, therapeutic framing, and caregiver and patient acceptance, reflecting the field’s maturation over the past three decades.

**Table 2:** Comparison of pre-2000 vs. 2000–2024 literature on therapeutic arts in Alzheimer disease.

Domain	Pre-2000 Literature	2000–2024 Literature
Study Designs	Small pilot trials, uncontrolled case series, variable methods.	RCTs, systematic reviews, meta-analyses, neuroimaging; greater standardization.
Sample Sizes	Very small samples (5–20 participants).	Moderate to large samples (30–200 participants).
Primary Focus	Mostly music therapy.	Broader: music, visual arts, dance/movement, expressive writing, digital arts.
Outcomes Measured	Behavioral (engagement, agitation), mood, social interaction.	Cognition, neuropsychiatric symptoms, QoL, caregiver burden.
Mechanistic Evidence	Minimal; mostly observational.	fMRI, EEG, biomarkers (melatonin, cortisol).
Reporting Quality	Inconsistent, non-standardized, no blinding.	Validated scales, structured protocols, some blinding.
Caregiver Attitudes	Viewed as recreational; limited institutional support.	Recognized as therapeutic; reduced caregiver stress, greater acceptance.
Patient Attitudes	Anecdotal; limited structured feedback.	Better documented PROs; improved mood, identity, and autonomy.
Institutional Adoption	Sporadic, staff-dependent.	Structured programs widely adopted globally.
Overall Evidence Strength	Encouraging but anecdotal.	Moderate evidence for behavioral and emotional benefits; mixed cognitive effects.

Abbreviations: RCT, Randomized Clinical Trial; QoL: quality of life; fMRI, Functional magnetic Resonance Imaging; EEG, Electro-encephalogram; PRO, Patient Reported Outcomes.

## Limitations of current evidence

Despite encouraging findings, the current evidence base remains limited by small sample sizes, heterogeneous intervention protocols, and short follow-up periods across studies. Blinding and allocation concealment are inherently difficult in arts-based interventions, which may introduce performance and detection bias. Historical studies published before 2000 further constrain interpretation due to inconsistent methodologies and the absence of standardized cognitive or behavioral outcome measures.

Additionally, the possibility of publication bias should be considered, as studies reporting positive outcomes may be more likely to be published, potentially overestimating the perceived benefits of therapeutic arts interventions. The cultural and socioeconomic context of these interventions also warrants attention, as access to structured arts-based programs, trained facilitators, and supportive environments may vary significantly across settings, influencing both feasibility and the generalizability of findings.

Furthermore, the use of objective neurobiological markers such as electroencephalography (EEG), functional magnetic resonance imaging (fMRI), or serum biomarkers remains sparse, primarily due to ethical and logistical constraints in vulnerable older populations. Larger, well-designed RCTs incorporating standardized protocols, longer follow-up, and neuroimaging-based assessments are needed to substantiate the therapeutic value of arts-based interventions in AD.

In conclusion, historical studies on therapeutic arts in AD were largely exploratory, involving small samples and predominantly music-based interventions. Over the past two decades, increasing methodological rigor has strengthened the evidence supporting their role as promising adjunctive non-pharmacological interventions, particularly for improving behavioral symptoms, emotional well-being, and social engagement.

However, given the heterogeneity across study designs, definitive evidence, especially regarding cognitive benefits in AD, remains inconclusive. Emerging neurobiological evidence suggests plausible mechanisms through which therapeutic arts may influence emotional processing, neural engagement, and quality of life, although these pathways require further validation in AD-specific populations.

While therapeutic arts are not a substitute for pharmacological treatment, they represent a safe, accessible, and person-centered approach that may complement multidisciplinary dementia care. Further standardized, large-scale studies are needed to better define their modality-specific effectiveness and long-term clinical relevance.

## Data Availability

No new data were generated or analyzed in this study.
